# Efficacy evaluation of some fumigants against *Fusarium oxysporum* and enhancement of tomato growth as elicitor-induced defense responses

**DOI:** 10.1038/s41598-023-29033-w

**Published:** 2023-02-11

**Authors:** Ahmed F. El-Aswad, Maher I. Aly, Sameh A. Alsahaty, Ayman B. A. Basyony

**Affiliations:** 1 Pesticide Chemistry and Technology Department, Faculty of Agriculture, Alexandria University, El-Shatby, Alexandria, 21545 Egypt; 2 Plant Pathology Department, Faculty of Agriculture, Alexandria University, Alexandria, Egypt

**Keywords:** Chemical biology, Plant sciences, Environmental sciences

## Abstract

Fusarium wilt, the most serious soil-borne pathogen, is a serious problem for tomato production worldwide. The presented study evaluated the antifungal activity against *Fusarium oxysporum *f. sp.* lycopersici *in vitro and in vivo for nine fumigants. In addition, the research examined the possibility of enhancing the growth of tomato plants in order to increase resistance against this disease by using four chemical inducers. The results indicated that at 20 mg/L, the radial growth of the pathogen was inhibited 100% by formaldehyde and > 80% by phosphine. Among the essential oils investigated, neem oil was the most effective, however, it only achieved 40.54% at 500 mg/L. The values of EC_50_ for all fumigants, except dimethyl disulfide (DMDS) and carbon disulfide (CS2), were lower than those for thiophanate-methyl. Phosphine was the highest efficient. The elicitors can be arranged based on their effectiveness, gibberellic acid (GA3) > sorbic acid > cytokinin (6-benzylaminopurine) > indole-3-butyric acid. The change in root length, fresh weight, and dry weight was greater with soil drench than with foliar application. The fumigant generators formaldehyde, phosphine and 1,4-dichlorobenzene and bio-fumigants citrus and neem oils as well as elicitors gibberellic and sorbic acid could be one of the promising alternatives to methyl bromide against *Fusarium oxysporum* as an important component of integrated management of Fusarium wilt.

## Introduction

The *Fusarium oxysporum* species are worldwide disseminated in indoor, soil, and marine habitats, and are economically destructive. *Fusarium oxysporum *f. sp. *lycopersici* commonly infects tomato crop and causes a disease known as Fusarium wilt, which is one of the limitations in tomato production areas. Consequently, it is one of the most prevalent and damaging diseases, because it is soil-borne, air-borne, and spread through plant residues^1,2^. The economic importance of *Fusarium oxysporum *f. sp.* lycopersici* derives from its destructive properties towards the tomato (*Solanum lycopersicum* Mill.), which is the most important horticultural crop in Egypt. It is widely cultivated throughout the year in three seasons on both old and new lands. Besides having a high nutritive value, tomato also has antioxidant and curative properties, making this vegetable crop the most consumed^1^.

Synthetic fungicides are widely used to control wilt diseases. Thiophanate-methyl was found to be effective against Fusarium wilt disease when applied as a soil drench and a seed dresser^3^. Nonetheless, soilborne pathogens (i.e., *Fusarium *spp.) are difficult to control with chemical fungicides. Unfortunately, the intensive application of chemical fungicides has adverse effects on plants, soil, environment, and human health, as well as development of fungicide resistance in pathogens^2,4^. Although the application of synthetic fungicides to tomato seeds reduces wilt incidence considerably, they are both costly and environmentally undesirable^5,6^. There is a great deal of concern about environmental contamination, as well as the emergence of fungi that are resistant to synthetic fungicides, prompting the search for new, safer alternatives. Hence, it is essential to develop alternative fungicides that are effective, environmentally friendly, and safe^2^. Fusarium wilt management using alternative methods may be an effective option for integrated management of this disease^7^.

The most effective management method for soil-borne wilt disease is soil fumigation. In light of the fact that methyl bromide (MeBr), which was highly effective to control various soil-borne pests, has been banned^8^, researchers have evaluated other alternatives such as chloropicrin, methyl iodide, metam sodium, and metam potassium. Until now, there have been no impressive alternate fumigants proven to be effective against Fusarium wilt, caused by *Fusarium oxysporum* f. sp. *lycopersici*, as demonstrated by methyl bromide^6^. Further, if infected seeds are transplanted into previously fumigated fields, the fumigation would have a slightly beneficial effect on controlling the disease^9^.

As phytochemicals are biodegradable, non-toxic, environmentally safe, they can be safely utilized against soilborne pathogens^10–14^. Essential oils are concentrated volatile hydrophobic liquids, extracted from aromatic plants. Certain essential oils have become great interest for their antimicrobial properties in both in vitro and in vivo, as well as their activity against foliar and soil-borne pathogens^15–17^. Due to the ability of some essential oils to be used with bio-control agents, they are considered as possible candidates for controlling Fusarium wilt diseases in field conditions^18^. Neem oil and garlic oil reduced the diameter of colonies and sporulation of *Fusarium oxysporum* in concentration-dependent manner^7^. However, the literature regarding the application of essential oils on *F. oxysporum *f. sp. *lycopersici* regarding Fusarium wilt diseases is scarce^4^.

An additional control strategy against Fusarium wilt is induced host resistance. Induced resistance is a non-specific form of disease resistance in plants acting against a wide-range of pathogens, and as such one would expect it to be activated by a range of non-specific inducers (elicitors), some have systemic activity, inducing resistance some distance away from the site of application, while others induce resistance locally at the site of application^19^. Elicitors induce plant resistance to pathogens, they are either small molecules or complex macromolecules, and either synthetic or natural. In addition, there are several different types of elicitors, classified as biotic or abiotic, general or race-specific, and exogenous or endogenous. The resistance inducers for controlling the diseases have been studied by several researchers in an attempt to minimize the use of pesticides, thus contributing to the advancement of sustainable agriculture^9^. Resistance can be achieved in many vegetable plants by applying various chemical inducers that have been found to increase growth parameters and fruits quality and quantity, such as salicylic, sorbic, and benzoic acids that have been found to be effective for enhancing resistance against several plant pathogens^20–22^. All known gibberellins are diterpenoids and are named from GA1 to GAn according to their discovery date. The first gibberellin to be structurally characterized was gibberellic acid (GA3). It is an organic substance naturally occurring in plants, it is a bioactive growth regulator and in fact essential to certain bioprocesses^23–25^. Indole-3-acetic acid is an inducer of resistance against phytopathogens, as well as known to be a plant growth regulator^26^. Since Indole-3-acetic acid has been detected to inhibit several phytopathogenic fungi, its role in controlling diseases may not be restricted to causing resistance in host cells but may also extend to the pathogen itself^27^. Thus, plant chemical resistance inducers could be commercially used to control tomato root rot diseases since they are safe, less expensive, and effective even in field conditions^28^.

Our aim is to identify effective strategies to control tomato wilt caused by *Fusarium oxysporum *f. sp. *lycopersici*. In this study we used an integrated approach and evaluated the fungicidal activity of various chemical and bio-fumigants as alternative to methyl bromide and tested the elicitation of induced resistance.

## Materials and methods

### Tested chemicals

#### Thiophanate-methyl

A commercial fungicide Tobist 70% WP was tested as a standard fungicide containing thiophanate-methyl, dimethyl 4,4′-(*o*-phenylene)bis(3-thioallophanate). It was obtained from the Egypt Chem Co., Egypt. It is systemic fungicide with protective and curative action. Water solubility 18.5 mg/L at 20 °C (pH 7), M.W. 342.4.

#### Fumigants

Three types of fumigants were tested.

##### True fumigants

Dimethyl disulfide (DMDS): Composition technical is ≥ 99% pure. M.W. 94.20, B.P. 109.6 °C, water solubility 2.7 g/L (20 °C). This compound under development as a soil fumigant. Carbon disulfide (CS_2_): It is a neurotoxic colorless volatile liquid. M.W. 76.13, 99.0% purity, water solubility 2.2 g/L (32 °C). Carbon disulfide is used as a fumigant for fumigation of nursery stock and for soil treatment against insects and nematodes.

##### Fumigant generators

Phosphine generators: Phosphine (PH_3_) is used as a fumigant, M.W. 34.0, B.P. − 87.4 °C, water solubility 26 cm^3^/100 mL at 17 °C. It was generated from Aluminum phosphide (AlP), Phostoxin Tablets 55% AlP, used for fumigation in stored products and in empty warehouses, silos, transport containers. M.W. 57.96 g/mol. Phosphine also was generated from Zinc phosphide (P_2_Zn_3_), Powder 80%, used as a bait rodenticide, M.W. 258.12 g/mol. 1,4-Dichlorobenzene (1,4-D): It is called Paradichlorobenzene, is an organic compound, a chlorinated aromatic hydrocarbon, MW 147 g/mol, B.P. 174 °C. It is a fumigant insecticide and repellent. It turns directly from a solid into a gas. The target organisms are molds, and insects. It is used as a fumigant to control clothes moths. Formaldehyde (HCHO): It can be used a fumigant fungicide and bactericide and used after cropping in glasshouses. It was obtained as a product from the reaction between formalin 40% with potassium permanganate. M.W. 30.0, B.P. − 19.5 °C, water solubility 55% (30 °C).

##### Biofumigants (essential oils)

Five essential oils were purchased from Pure Life Co., Cairo, Egypt. All samples were stored in hermetically sealed vials at 4 °C in the dark until use. Oil of jojoba *Simmondsia chinensis*, density 0.863–0.873 g/cm^3^. Its composition in fatty acids is palmitic acid 35%, oleic acid 15%, gadolic acid 65–80%, erucic acid 10–20%. Oil of garlic A*llium sativum*, density 0.863–0.873 g/cm^3^. Its composition in fatty acids is palmitic acid 10–14%, oleic acid 20–30%, palmitoleic acid 1%, stearic acid 4%, linoleic acid 5–6%, arachidic acid 4%, linolenic acid 1%, behenic acid 3.5%, gadolic acid 1%, and erucic acid 1.5%. Active ingredients in oil daily disulfide 3%, daily trisulfide 24–37%, and allicin 39–50%. Oil of argan *Argania spinose*, density 0.888–0.898 g/cm^3^. Its composition in fatty acids is saturated fat is 10%, mono- unsaturated fat 73%, poly unsaturated fat 1%, fatty acids omega-6 3.6%. Oil of neem *Azadirachta indica*, density 0.900–0.920 g/cm^3^. Its composition in fatty acids is palmitic acid 15.59%, oleic acid 41.91%, palmitoleic acid 12%, stearic acid 18.71%, linoleic acid 19.59%, arachidic acid 1.33%, linolenic acid 0.44%, behenic acid 0.86%, gadolic acid 0.08%, and saturated fatty acids 37%, unsaturated acids 63%. Active ingredients in oil tocopherols composition δ-tocopherol 11.13%, β tocopherol 0.59%, υ-tocopherol 68.88%, and α-tocopherol 19.38%. Oil of orang *Citrus sinensis*, density 0.858–0.890 g/cm^3^. Active substance limonene 93.7%, α-pinene 0.65%, sabinene and β-pinene 1%, myrecen 2.09%, octanal 0.41%, linalool 0.31%, δ-3-carene 0.31%, and dicanal 0.27%.

#### Plant resistance elicitors

Four chemical elicitors were tested. Gibberellic acid (GA3) as Gibbrolex top TB 10%, supplied by Chem Co. Egypt. Indole-butyric acid (IBA) as Life Indole 2% WP and Cytokinin (6-benzyl amino burien) as Life Mark WP 8%, obtained from Damak Co. Egypt, Sorbic acid 100% was purchased from Elgomhoria Co. Egypt.

### Tested plant

Tomato (*Solanum lycopersicum *L.) is F1 Dussehra hybrid variety, produced by Kanza group for projects development and services. Tomato seedlings 30 days old were obtained from the Agricultural Research Centre (ARC), Egypt. The seedlings were transplanted in pots (16 cm diameter, 20 cm height) containing sterilized soil (un-infested or fungal infested soil) at the rate of 1 seedling/500 g soil/pot. A set of three pots were used for each treatment as replicates, as well as, for the un-infested soil as control.

### Tested soil

Soil samples were taken from the top 15 cm in a field at Agricultural Experiment Station, Faculty of Agriculture, Alexandria University. Soil samples have been no history of pesticide or fumigation treatment. Soil analysis indicated that the soil texture was sandy loam soil (30% clay, 44% silt, 26% sand), pH 8.1, EC 680 ppm, organic carbon content 0.92%, HCO_3_^−^ 245 ppm and total cations 338 ppm. The field soil samples were passed through a 4-mm sieve and stored at room temperature for 48 h before being used in the experiment.

### Tested pathogen

#### Isolation and identification of the fungus

The soil-borne pathogen *Fusarium oxysporum* f. sp. *lycopersici* was isolated from soil and root sample of naturally infected tomato plants showing typical wilt disease symptoms^29^. The samples were collected from the fields from El Nubaria, Beheira Governorate, Egypt. Under sterilize condition, roots were washed under tap water, chopped into 2 cm pieces, and surface sterilized in NaOCl (0.5%) for two minutes, then rinsed twice with distilled water. Roots were then placed on Potato dextrose agar (PDA) and kept in an incubator at 27 °C under dark conditions^30^. After 5 days of incubation, the Cork borer was used to cut the PDA culture covered by the fungus mycelium to discs (5-mm diameter), five discs were transferred to fresh PDA dishes (9 cm diameter) containing 9 mL of PDA medium. Dishes were incubated at 25 °C for 7 days. *Fusarium oxysporum *f. sp. *lycopersici* was identified by Plant Pathology Department, Faculty of Agriculture, Alexandria University according to the manual^31^. Fungal mass production was obtained by growing the fungus on sterilized barley grain. Conical flasks contained sterilized barely grain, were autoclaved at 121 °C, 1.5 kg/cm^2^ for 30 min twice. The autoclaved flasks were inoculated by the fungus (10 mycelial discs of the fungus medium to 250 mL conical flask); and incubated at 25 ± 2 °C for 2 weeks^2,32^.

#### Pathogenicity test

Pathogenicity test was carried out using the method of Haggag and El-Gamal^33^ to investigate the pathogenic potentiality of the isolated fungus. The infested barely grain was thoroughly mixed with autoclaved soil at the rate of 3% of soil weight. Four-weeks-old tomato seedlings were transplanted into the infested soil (one seedling per pot). The disease incidence was determined by recording the percentage of dead seedlings, 45 days after transplanting.

### Experimental design

#### In vitro antifungal activity assay

The in vitro antifungal activity of different fumigants was evaluated on *Fusarium oxysporum* using mini-atmosphere and poisoned food methods.

#### Mini-atmosphere method

The in vitro effect of bio-fumigants at 250 and 500 mg/L of space and fumigant generators at 10 and 20 mg/L of space, was carried out using mini-atmosphere method described by^34,35^, which allows to study the effect of the volatile compounds.

A disk (5 mm in diameter) from 7-days-old Fusarium culture on PDA medium was transferred to the center of the conical flask contained 9 mL PDA medium. A sterilized cotton pieces were impregnated with known volume of the essential oil solutions in dimethyl sulfoxide (DMSO), the amounts equivalent to concentrations of 250 and 500 mg/L of the air inside the flasks. Also, only DMSO was used as a control to confirm no effect of the solvent on the bioactivity. The cotton pieces were hanged into the flasks. Also, small vials contained the source of a fumigant were hanged into sealed conical flasks. The source of phosphine was AlP or Zn_3_P_2_ mixed with HCl 1 N (1:5 ratio) to accelerate release of gas and the source of formaldehyde was formalin 40% mixed with KMnO_4_. After treatment, the flasks were incubated at 25 °C. Inhibition of the mycelial growth was estimated at 7th day of incubation by measuring radial growth of the fungus exposed to different concentrations of the fumigants and compared to the control which had full growth. Five replicates per treatment were used and the experiment was repeated twice. The antifungal activity was expressed in terms of percentage of mycelial growth inhibition and calculated according to the following formula:

$$ {\text{GI}}\;(\% ) = [({\text{Gc}} - {\text{Gt}})/{\text{Gc}}] \times 100 $$

where GI % is the percentage of Mycelial Growth Inhibition, Gc is the growth as a mycelium radius of the negative control and Gt is the mycelium radius of the treatment^36^.

#### Poisoned food assay method

Antifungal activity of true- and bio- fumigants on radial growth of *Fusarium oxysporum* was determined using the poisoned food technique^2,37^. Known quantities of DMDS, CS_2_ and essential oil solutions contained TritonX-100 and DMSO, were incorporated into the sterilized and melted PDA medium, to obtain 250 and 500 mg/L of medium. About 9 mL of the medium were poured into sterile 9 cm diameter Petri dishes. Subsequently, inoculated at the center with a mycelial disc (5-mm diameter) taken from the actively growing of 4–6 days old Fusarium culture. The disk was placed with the mycelium side down^38^. Two types of control without essential oil were inoculated following the same procedure. The negative containing PDA treated with sterile distilled water and the positive control containing PDA plus aliquots of surfactant and solvent. The Petri dishes were sealed and incubated in the dark at 25 ± 2 °C for 7 days. Also, thiophanate-methyl (100 mg/L) was added to PDA medium before solidifying and rotated gently to ensure an even distribution of fungicide. Five replicates were used for each concentration. The mycelial diameter was daily measured until the negative control reached the edge of the dish. Efficacy of treatments was recorded as a percent reduction of *F. oxysporum* growth compared with the untreated control.

#### In vivo antifungal activity assay

Effect of fumigants on *Fusarium oxysporum* in infested soil was evaluated. The experiment was carried out in the greenhouse of Plant Pathology department, Faculty of Agriculture, Alexandria University.

#### Soil infestation

The samples (250 g) of soil were placed into PVC jars (8 cm diameter, 16 cm height, circular cover for sealing). The soil samples were artificially infested with 1-week old inoculum of Fusarium grown on barley at the rate 3% w/w of soil weight. Three replicates per treatment and three sub-samples for each sample were used and the experiment was repeated twice. Three jars containing infested soil were served as an infested control. Jars were kept at 25–28 °C, 70–80% relative humidity and 12 h photoperiod and watered as needed.

#### Fumigant treatment

The efficacy of the fumigant generators using mini-atmosphere method was investigated. Infested soil samples were exposed to fumigant generators at concentrations of 1.0, 2.5, 5.0, 7.5, 10.0, 25.0, 35.0, 50.0 mg fumigant gas/L of space. Soil drench method was used for treatment of true- and bio-fumigants and thiophanate-methyl. The soil samples were treated with 25 mL of different fumigant solutions to obtain concentrations of 15, 30, 45, 50, 65, 80, 100 and 120 mg fumigant/kg soil). Essential oil solutions were emulsified with Triton-X100^39^. After treatment, jars were sealed and kept under greenhouse conditions. For fungal community analysis, three soil samples (1 g of each) were taken from each replicate (9 samples for treatment) at 1 week after treatment and mixed with PDA medium in Petri dishes.

#### Elicitation experiment

The greenhouse experiment was done to evaluate the efficacy of tested elicitors according to the procedure described by^40,41^. Four chemical resistance inducers gibberellic acid (GA3), indole-butyric acid (IBA), sorbic acid (SoA), and cytokinin 6-benzyl amino burien (Cytok) were evaluated at four concentrations 12.5, 25.0, and 50.0 mg/kg, equivalent to 0.5, 1, and 2 folds of the recommended rate. Thiophanate-methyl was applied at 50 mg/kg equivalent to its recommended rate.

#### Soil infestation

Sterilized soil in pots (16 cm diameter, 20 cm height) was artificially infested with 1 week inoculum of Fusarium grown on barley at the rate 3% w/w of soil weight.

#### Cultivation of tomato seedlings

Four weeks old of healthy tomato seedlings were transplanted into the pots containing infested soil (1 seedling/500 g/pot). Healthy control (no Fusarium inoculation and no elicitors), infested control (with Fusarium inoculation and no elicitors), and treatments (with *Fusarium* inoculation and treated with elicitors), each has five replicates, were tested.

#### Treatment of tomato plants with elicitors

Four elicitors used in the elicitation experiment were GA, SoA, IBA and Cytok. Elicitors were dissolved in sterile distilled water to obtain 125, 250, and 500 mg/L concentrations. For soil drench method, 25 mL of each concentration was applied to soil at 7 days after transplanting to obtain final concentrations of 12.5, 25.0, and 50.0 mg/kg soil. The moisture of soil will be 60% of the water holding capacity of soil. For foliar application, the tomato plants were foliar sprayed with elicitor at concentration of 250 mg/L (the recommended rate) at 7 days after transplanting. Treatments were kept in the greenhouse under 25 °C receiving water as required. Phytotoxicity of the elicitors were monitored. After 60 days, the plant samples were collected carefully for plant growth measurements.

#### Assessment of elicitors efficacy

After 60 days of treatment, tomato plants were manually removed from their pots after being flooded with water in order to not damage their roots. Shoot and root lengths, as well as fresh and dry weight of the plant, were measured as growth parameters. The effectiveness of chemical resistance inducers was determined by comparing them with infected control using the following equations:

$$ {\text{Efficacy}} \% {\text{as fresh or root enhancement}}=\left(\frac{{\text{Shoot or root length in treatment}}}{{\text{Shoot or root length in control}}}\right) \times 100$$

$${\text{Efficacy}} \% {\text{based on fresh or dry weight}}=\left(\frac{{\text{Fresh or dry weight in treatment}}}{{\text{Fresh or dry weight in control}}}\right) \times 100$$

### Statistical analysis

Data of the fumigation and elicitation experiments are presented as mean ± standard deviation, and the statistical analysis was performed using the SAS software (SAS Institute Inc., Cary, NC, USA). One-way analysis of variance (ANOVA) was used to analyze the data of growth inhibition by fumigants and growth induction by elicitors. The Least Significant Difference (LSD) test at P ≤ 0.05 was used for mean comparison. Data of in vivo assay were analyzed by Finny test to obtain the EC_50_^42^.

### About the experiments of tomato plants

I would like to confirm that all methods were performed in accordance with the relevant guidelines and regulations.

## Results

### Identification of the isolated fungus and its pathogenicity potential

The soil-borne pathogen isolated from root sample of naturally infected tomato plants grown in infested region has long history with *Fusarium oxysporum *f. sp. *lycopersici*, showing typical wilt disease symptoms according to Davet and Rouxel^29^. *Fusarium oxysporum* f. sp.* lycopersici* was identified by Plant Pathology Department, Faculty of Agriculture, Alexandria University, based on the cultural properties, morphological and microscopic characteristics according to the manual^31^. The pathogenicity test indicated that the isolate of fungus has ability to cause wilt disease of tomato plants. At 45 days after transplanting, the symptoms are wilt symptoms on tomato leaflets and vascular discoloration in the xylem vessels. The disease incidence was determined by recording the percentage of dead seedlings, it was 80%. This result is in harmony with those reported by Thabet and Khalifa^1^.

### Effect of fumigants on growth of *Fusarium* in vitro after 7 days exposure

Effect of fumigants against *Fusarium oxysporum* grown on PDA medium after 7 days exposure was investigated. All fumigants tested significantly reduced the radial growth of Fusarium in vitro compared with control, and the reduction in radial growth increased with increasing concentrations (Table 1). The fumigant generators were the highest effective compounds at the tested concentrations compared to true- and bio-fumigants. Using mini-atmosphere method, the radial growth of the pathogen was fully inhibited (100%) by formaldehyde at 20 mg/L of space. A phosphine gas generated from zinc phosphide and aluminum phosphide inhibits radial growth of pathogen by 63.68 and 61.08% at 10 mg/L, and by 80.38 and 81.68% at 20 mg/L, respectively. DMDS and CS_2_ both had weak effects on *F. oxysporum* (< 35% inhibition) at 500 mg/L, however, CS_2_ found to be twice as effective as DMDS. Among investigated essential oils using poisoned food method, the most effective was neem oil, however it achieved only 27.03 and 40.54% at 250 and 500 mg/L of medium, respectively. While the same concentrations of the other oils showed less inhibition (< 40%) (Table 1). Furthermore, the effect of volatile phase of the essential oils using mini-atmosphere method on the fungus within 7 day-exposure was negligible. Growth inhibition of Fusarium on PDA media containing 100 mg/L of fungicide thiophanate-methyl was 100%. Surfactant (Triton-X100) and DMSO that used in essential oil aqueous solutions showed only 2.70% radial growth inhibition (Table 1).

**Table 1:** Effect of tested true- and bio-fumigants using food poisoned method and fumigant generators using mini-atmosphere method against Fusarium grown in PDA medium at 7th day.

Treatments		Mean of radial growth (mm)^1^	% Growth inhibition^2^ ± SD		
Control^3^	(−)	617	0.00 ± 0.14		
	(+)	583	2.70 ± 0.28		
Thiophanate-methyl (100 mg/L)		0	100.00 ± 0.00		

Food poisoned method					
		250 mg/L of medium	500 mg/L of medium	250 mg/L of medium	500 mg/L of medium
Bio-fumigants (essential oils)	Garlic	483	383	21.62 ± 0.29	37.84 ± 0.29
	Jojoba	467	417	24.32 ± 0.29	32.43 ± 0.44
	Citrus	500	450	18.92 ± 0.15	27.03 ± 0.71
	Neem	450	367	27.03 ± 0.00	40.54 ± 0.29
	Argan	617	500	0.00 ± 0.29	18.92 ± 0.76
True fumigants^4^	DMDS	550	500	10.81 ± 0.29	18.92 ± 0.15
	CS2	450	417	27.03 ± 0.00	32.43 ± 0.29

Mini-atmosphere method					
		10 mg/L of space	20 mg/L of space	10 mg/L of space	20 mg/L of space
Fumigant generators	Zn-phosphide atmosphere method against Fusarium^5^	224	121	63.68 ± 1.40	80.38 ± 0.15
	Al-phosphide^6^	240	113	61.08 ± 0.71	81.68 ± 1.50
	1,4-D^7^	338	207	45.19 ± 1.79	66.43 ± 0.29
	Formaldehyde^8^	139	0	77.46 ± 0.44	100.00 ± 0.00

^1^The values represent the mean of the 5 replicates.

^2^Growth inhibition (%) = ((Growth in control − Growth in treatment)/Growth in control) × 100.

^3^Control (−) containing water, control (+) containing (1 mL Triton-X 100 + 1 mL DMSO)/L of medium.

^4^Dimethyl disulphide (DMDS), Carbon disulphide (CS_2_).

^5^Zn-phosphide was mixed with HCl 1N (1:5), 1 mg Zn-phosphide produces 0.24 mg PH_3_.

^6^Al-phosphide was mixed with HCl 1 N (1:5), 1 mg AL-phosphide produces 0.32 mg PH_3_.

^7^It was generated from 1,4-dichlorobenzene (100%).

^8^Formaldehyde was generated from formalin 40% + KMnO_4_ (1:3), 1 mL formalin produces 0.40 g formaldehyde.

### Effect of fumigants on fusarium in soil under greenhouse conditions

The fungicidal efficiency of eleven fumigants and chemical fungicide thiophanate-methyl at various concentrations against *F. oxysporum* in soil was evaluated. The soil drench method for true-and bio-fumigants and mini-atmosphere method for fumigant generators were used under greenhouse conditions. The data revealed that the growth inhibition of Fusarium increased linearly with an increase in concentration of all treatments. The statistical analysis showed that tested fumigants significantly affected (P ≤ 0.05) mycelial growth of the fungus. In terms of their effectiveness, fumigants could be categorized as three groups based on their EC_50_. The first group, which was very efficient, included phosphine (Al-phosphide and Zn-phosphide). The second group that had a high effect included 1,4-D, formaldehyde, and essential oils. The third group, which had a moderate effect, included true-fumigants (DMDS and CS_2_) as well as fungicide thiophanate-methyl. There were significant differences in efficacy between phosphine and 1,4-D or between phosphine and formaldehyde, but no significant differences were detected between the fumigant generators 1,4-D and formaldehyde or between both true-fumigants DMDS and CS_2_ (Table 2). On the basis of their EC_50_ values, the essential oils can be arranged as follows: citrus (high effective) < neem < argan < jojoba < garlic. Using the mini-atmosphere method, however, the essential oils had no effect on the fungus in the soil. As demonstrated in Table 2, thiophanate-methyl was significantly less effective than all of the fumigants except DMDS and CS_2_.

**Table 2:** Effect of tested fumigants on fusarium in soil using drench method for true- and bio-fumigants and mini-atmosphere method for fumigant generators under greenhouse conditions.

Treatment	EC_50_^1^	Slop	Chi^2^
Soil drench method, concentration in mg/kg soil			
Thiophanate-methyl	54.0^e^ (62.6–46.8)	2.041 ± 0.025	28.820
Bio-fumigants (essential oils)			
Garlic	52.6^de^ (68.0–39.8)	1.047 ± 0.005	2.990
Jojoba	43.0^d^ (62.0–27.8)	0.710 ± 0.004	8.578
Citrus	19.2^c^ (32.8–9.4)	0.626 ± 0.004	13.751
Neem	32.6^c^ (43.2–23.6)	1.015 ± 0.006	3.336
Argan	39.2^ cd^ (53.2–27.4)	0.880 ± 0.005	0.919
True fumigants^2^			
DMDS	98.6^ef^ (129.4–73.0)	1.128 ± 0.006	0.570
CS_2_	56.4^e^ (76.2–40.2)	1.007 ± 0.005	2.027
Mini-atmosphere method, concentration in mg/L of space			
Fumigant generators			
Zn-phosphide^3^	5.5^a^ (6.7–4.6)	1.620 ± 0.002	9.695
Al-phosphide^4^	5.2^a^ (7.5–3.6)	1.424 ± 0.026	14.243
1,4-D^5^	24.0^b^ (56.0–10.0)	1.128 ± 0.057	3.535
Formaldehyde^6^	39.6^b^ (54.0–28.0)	2.019 ± 0.074	13.878

^1^Median effective concentration calculated from growth inhibition data, values marked by the different letters in the column indicate a significant difference at P ≤ 0.05.

^2^Dimethyl disulphide (DMDS), Carbon disulphide (CS_2_).

^3^Zn-phosphide was mixed with HCl 1N (1:5), 1 mg Zn-phosphide produces 0.24 mg PH_3_.

^4^Al-phosphide was mixed with HCl 1N (1:5), 1 mg AL-phosphide produces 0.32 mg PH_3_.

^5^It was generated from 1,4-dichlorobenzene (100%).

^6^Formaldehyde was generated from formalin 40% + KMnO_4_ (1:3), 1 mL formalin produces 0.40 g formaldehyde.

### Effect of chemical resistance inducers on the growth parameters of tomato plants

It is very important to state that during 60 days after elicitor treatments via soil drenching, none of the elicitors, as well as fungicide thiophanate-methyl, caused any phytotoxicity in tomato plants. The results showed that all tested elicitors at 25 and 50 mg/kg soil had a significant positive effect on the shoot and root length of tomato plants in comparison to infested control plants (Table 3). The highest tomato plant growth enhancement was found to be 0.5 and 0.3-folds for shoot length and 3 and 2.8-folds for root length with GA and SoA (both at 50 mg/kg soil). However, at the lowest concentration (12.5 mg/kg), IBA and GA reduced shoot length to 72.0% and 96.0%, respectively, compared to infested control (100%).

**Table 3:** Effect of chemical resistance inducers on growth parameters of tomato plants grown in Fusarium infested soil under greenhouse condition.

Treatment		Concentration		% Effectiveness			
Trade name	Active ingredient	mg/kg soil	Fold of rate	Shoot length	Root length	Fresh weight	Dry weight
Control (infested)			–	100.0^ij^	100.0^j^	100.0^h^	100.0^g^
Control (healthy)			–	138.7^b^	195.0^g^	329.5^a^	286.5^a^
Tobist 70%	Thiophanate-methyl	50	1	136.0^bc^	180.6^gh^	145.6^g^	154.9^f^
Life mark (8%)	Cytokinin (6-benzylaminopurine)	12.5	0.5	120.0^g^	300.0^d^	218.6^de^	192.5^def^
		25.0	1	124.0^efg^	325.0^c^	274.5^bc^	268.3^a^
		50.0	2	120.0^g^	300.0^d^	240.0^cd^	248.8^abc^
Indole butyric acid (100%)	Indole butyric acid	12.5	0.5	72.0^k^	133.3^i^	147.9^fg^	168.8^f^
		25.0	1	122.7^fg^	175.0^gh^	227.1^d^	219.2^bcd^
		50.0	2	105.7^hi^	164.7^h^	226.0^d^	212.3^cde^
Sorbic acid (100%)	Sorbic acid	12.5	0.5	109.3^h^	225.0^f^	184.0^ef^	172.3^ef^
		25.0	1	128.0^def^	333.3^c^	320.7^a^	280.9^a^
		50.0	2	133.3^bcd^	383.5^ab^	273.4^b^	265.0^a^
Gibrilex (10%)	Gibberellic acid	12.5	0.5	96.0^j^	275.0^e^	210.6^de^	180.8^def^
		25.0	1	130.7^cde^	366.7^b^	283.2^b^	271.7^a^
		50.0	2	147.0^a^	396.5^a^	271.2^bc^	258.2^abc^

Means in the column followed by different letters indicate significant differences among treatments at P ≤ 0.05.

GA and SoA at 25 mg/kg were more effective than at 12.5 mg/kg, but less effective than at 50 mg/kg soil. While the effect of Cytok and IBA at 25 mg/kg (equivalent to the recommended rate) was greater than that at 50 mg/kg (2-folds) and 12.5 mg/kg (0.5-fold). No significant differences were detected in the effectiveness of elicitors at 25 mg/kg on shoot length. The healthy control and the thiophanate-methyl treatment showed the highest efficiency on shoot length and the lowest efficiency on root length other than GA at 50 mg/kg and IBA at all tested concentrations. Meanwhile, there are no significant difference in shoot length and root length between treated plants by thiophanate-methyl and untreated plants in the healthy control (Table 3). On the basis of the statistical analysis of their effects on shoots and roots, the different treatments could be divided into different groups, in the following order: (GA at 50 mg/kg) > (SoA at 25 and 50 mg/kg, GA at 25 mg/kg) > (IBA at 25 mg/kg, Cytok at all concentrations) > (SoA at 12.5 mg/kg, GA at 25 mg/kg) > (IBA at 12.5 and 50 mg/kg soil).

Comparing to the infested control, all elicitors at different concentrations significantly increased the fresh and dry weights of tomato plants (g/plant), where the enhancement ranged from 2.2 to 0.5-folds and from 1.8 to 0.7-folds, respectively (Table 3). Also, thiophanate-methyl increased the fresh weight by 45.6% and the dry weight by 54.9%. In addition, all elicitors at 25 and 50 mg/kg soil increased fresh and dry weights of tomato plants more than those at 12.5 mg/kg soil (Table 3). All elicitors were more effective at 25 mg/kg (1F) than they were at 50 mg/kg (2F), followed by that at 12.5 mg/kg (0.5F).

### Effect of treatment method on elicitors efficiency

**Figure 1:**
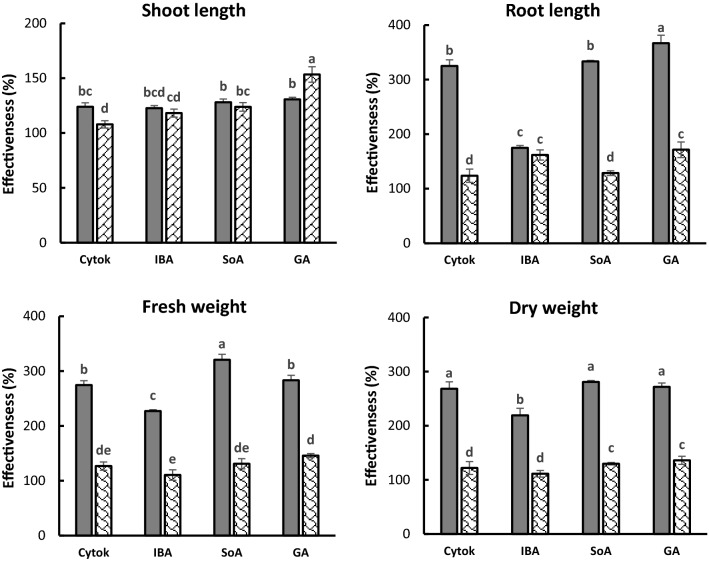
Comparison between effect of chemical resistance inducers at their recommended rate applied by soil drench  and foliar spray on growth parameters of tomato plants grown in Fusarium infested soil under greenhouse condition. *Cyto* cytokinin, *IBA* indole butyric acid, *SoA* sorbic acid, *GA* gibberellic acid. Different letters indicate significant differences among treatments in each growth parameter at P ≤ 0.05.

The extent to which different treatment methods of elicitors application as soil drench or foliar spray using the recommended rates, affected enhancement of growth parameters of tomato plants was examined. For all elicitors applied either by soil drench or by foliar application during the experiment period, no phytotoxicity was detected in tomato plants. Figure 1 shows the soil treatment had a significant effect on the root length, fresh weight and dry weight of tomato plants, exceeding that exhibited by foliar application of all elicitors. For SoA and IBA, there were no significant differences in shoot length between the two methods of soil drench and foliar spray. As shown in Fig. 1, GA and SoA were more effectiveness on different tomato growth parameters than Cytok and IBA, using both soil applied and foliar spray methods. Also, the effect of Cytok was more than that of IBA with soil drench method. Furthermore, it was observed that the shoot length was less susceptible for elicitors compared to other growth parameters. In addition, the soil treatment method showed a significantly greater change in root length than foliar treatment. In contrast, the foliar spray showed high effect on the shoot length. Consequently, the difference in effect of elicitors between the two treatment methods was very narrow on shoot length while it was very wide on the other parameters of tomato growth.

## Discussion

Soilborne diseases including root rots are involved in considerable losses of the most important vegetable crops. In the light of the economic importance of tomato, it is extremely promising that protective products with lower risks to human health and the environment can be used in controlling *Fusarium oxysporum* wilt disease^43^. In order to maintain quality and quantity of tomato fruits, Fusarium wilt should be controlled. However, this disease is difficult to control, according to^44^. In general, the effectiveness of fungicides against soil-borne pathogens is lower than their efficacy against aerial pathogens. As well, non-target effects, chemical resistance, environmental risks and the phasing out of many active compounds for environmental reasons, for example methyl bromide, have contributed to the search for alternatives to be used to treat wilt disease^6,45^. It has been shown that Fusarium species are generally reduced by soil fumigation^46^, although they are less sensitive to certain fumigants.

According to our results, the tested fumigant generators (phosphine, 1,4-D, and formaldehyde) showed higher inhibition, whereas true-fumigants (DMDS and CS_2_) caused lower effects compared to bio-fumigants on Fusarium grown on PDA medium or in soil. It is very interesting to observe that the effect of phosphine based on the concentration rather than on its generation source. Formaldehyde was the highest effective fumigant for in vitro assay using PDA medium. It had also previously been reported to have high antimicrobial effects^47^. The cytotoxicity of formaldehyde is due to its reactivity as an electrophile, were reacts rapidly with free thiol (–SH) and amine (–NH_2_) groups, causing DNA and protein damage^48–50^. In addition, the most effective fumigant for fungus grown in soil was phosphine gas emitted by AlP or Zn_3_P_2_. In contrast, it was found that DMDS was the least effective compound against Fusarium grown in soil or on PDA medium. This is in agreement with a reported by^51^ who found that DMDS had a relatively weak effect on *F. oxysporum*. In general, using food poisoned or mini-atmosphere method, Fusarium grown in soil or on PDA medium exhibited growth inhibition in a concentration dependent manner. On PDA medium, thiophanate-methyl was more effective than true-fumigant and bio-fumigant against *F. oxysporum*. While, in soil, thiophanate-methyl was less effective than all fumigants except DMDS and CS_2_. Also, the efficacy of formaldehyde on *F. oxysporum* reduced in the case of in vivo assay compared to in vitro assay. The results could be explained by the adsorption of the fungicide on the soil particles. Recent studies have shown that thiophanate-methyl is effective in lowering Fusarium wilt^52^.

In terms of essential oils, neem oil had the strongest growth inhibition (40.54%) at 500 mg/L concentration, followed by garlic oil, which was comparatively less effective (37.54%), while argan oil showed the least growth inhibition (18.92%). As for the fungicidal activity against *F. oxysporum* in soil, the essential oils might be arranged according to their EC_50_ values; citrus (106 ppm) > neem (163 ppm) > argan (196 ppm) > jojoba (215 ppm) > garlic (263 ppm). The high effectiveness of neem oil in this study is consistent with other studies. Neem extracts have been revealed to inhibit microbial activity^53^. Also, neem oil at various concentrations inhibited mycelial growth of *Fusarium oxysporum* on PDA^7,54^. In neem oil, azadirachtin, which belongs to the C25 terpenoids family, has been found to be the most important antifungal compound^55^. There is a significant difference in the fungicidal activity of essential oils, this difference could be caused by various compounds in oils^56,57^. While some oils have a simple profile, others contain a wide variety of chemical compounds^58^. Essential oils possess antifungal properties because of their phenolic compounds^59^. Moreover, the laboratory and greenhouse experiments revealed that the effect of essential oils applied on *F. oxysporum* was higher using food poisoned method than that of mini-atmosphere method, where its effect was negligible. Furthermore, the essential oils influenced the fungus in the soil more than the fungus on the PDA medium, this might be due to direct contact of the oil solution with the fungus grown in the soil because the oil was applied on the 7th day following soil infestation. Generally, essential oils have been reported to be one of the most promising groups of natural compounds for the development of antifungal agents that are safe for the environment to use in managing diseases^57^.

As a whole, the results indicated that phosphine, 1,4-D, and formaldehyde, as well as essential oils, are promising compounds for the integrated management of wilt disease. Although, precautions and the cost of application need to be considered. Also, the effects of fumigating these compounds must be studied. Studies conducted on soil microorganisms over a short period have found that fumigation does not negatively affect non-target organisms. However, a number of studies suggest that most fumigants also kill beneficial microorganisms^60,61^. Therefore, regardless of whether a short-term or long-term study is conducted, the residual effects of various fumigants on beneficial microorganisms remain a mystery^62^.

### Effect of chemical resistance inducers on growth parameters of tomato plants

Resistance inducers are often used as bioactive substances in soilborne and foliar plant disease control^21,63–65^. As part of this study, soil treatments of GA, Cytok, IBA and SoA at different concentrations were tested for their efficacy in improving tomato plant growth to protect plants against root rot caused by Fusarium in artificial soil under greenhouse conditions.

Soil infested by *F. oxysporum* significantly decreased shoots and roots length and fresh and dry weights of tomato plants. A study also showed that growth of shoots and roots was significantly inhibited in tomato plants with Fusarium wilt, in comparison with healthy controls^66^. Perhaps this is a result of fungi producing toxins that affect K^+^ uptake and stomata function, causing excessive transpiration and water loss, leading to weak plants^67^. As a result of application of elicitors, significant improvements were found in the growth parameters of tomato plants, indicating that it can enhance resistance to Fusarium wilt. In the presence of pathogenic fungus, chemical fungicide thiophanate-methyl at 50 mg/kg soil (equivalent to onefold the recommended rate) as well as all elicitors at 25 and 50 mg/kg soil (1F and 2F) significantly enhanced the growth parameters of tomato plants. Those findings are in agreement with previous research reports^22,65,68–70^, which demonstrated that many chemical resistance inducers were effective against root rot pathogens infecting many crops. Furthermore, IBA applied at 1F increased all growth parameters of tomato plants more than IBA applied at 2F. This finding is consistent with prior findings^71–74^. They found that IBA applied at higher concentrations beyond the optimum was inhibitory. Therefore, the growth regulators have to be applied in the appropriate concentrations. However, it is worth noting that the optimal concentration of IBA varies with the species of plants^75^.

On the basis of the effectiveness on tomato growth parameters, the elicitors at all concentrations could be arranged as follows: GA > SoA > Cytok > IBA. It has been suggested that the positive effects of chemical inducers on tomato plant growth may be due to their effects on physiological processes within the plant such as ion uptake and transport, cell elongation, cell division, enzymes activation, protein synthesis, membrane permeability, and photosynthesis^64,76,77^. Moreover, Cytok has an effect on root and shoot meristem activity as well as plant development^78–80^. Plants are induced with an elicitor dependent signaling cascade that leads to a systemic expression of long-lasting disease resistance that is efficient against fungi, bacteria, and viruses^81^. Additionally, the chemical inducers may activate defense mechanisms such as phenolic compounds, oxidative enzymes and other metabolic pathways^20,63,82,83^. Similar to that found by other researchers^84^, the elicitors applied as a soil drench or foliar spray can activate the growth parameters that reduce Fusarium fungal infection causing wilt to tomato plants, however, soil drench significantly enhanced the plant responses more than the foliar spray as well as the untreated inoculated control. Thus, today, in order to move toward a sustainable agriculture and reduce the use of pesticides and chemical fertilizers, the elicitors with positive effects should be used^85^.

## Conclusion

In the present study, fumigant generators formaldehyde, phosphine and 1,4-D as well as chemical resistance inducers GA and SoA displayed promising efficiency as better alternatives to MeBr in the management of Fusarium. Therefore, under the umbrella of Integrated Disease Management (IDM) for tomato crop, it is suggested that soil fumigation by effective fumigants (phosphine or 1,4-D at 20 mg/L of space) is applied pre-plant in order to control soil-borne diseases and effective chemical resistance inducers (gibberellic acid or sorbic acid at 25 mg/kg soil) are applied to soil via drenching to improve plant growth for increased resistance. There remains a needed for further research on the long-term effects of these alternative fumigants on soil health and beneficial microorganisms in order to select fumigants that have low adverse environmental impacts, maintain soil health, and improve sustainable crop production. This study also demonstrated that the essential oils citrus and neem oils could be a good alternative synthetic fungicide as well as will be better alternative to MeBr, as they are more environmentally friendly and known to have minimal danger to consumers. Therefore, it can be applied in organic farming for the management of *Fusarium oxysporum*. Nevertheless, economic assessment is required to enhance their application.

## Data Availability

The datasets used and/or analysed during the current study available from the corresponding author on reasonable request.
